# Flow cytometry and cell sorting

**DOI:** 10.3389/fmed.2023.1287884

**Published:** 2023-11-22

**Authors:** William G. Telford

**Affiliations:** Laboratory of Pathology, National Cancer Institute, National Institutes of Health, Bethesda, MD, United States

**Keywords:** flow cytometry, cell sorting, electrostatic, nozzle, flow cell, microfluidics, drop delay

## Abstract

While flow cytometry is a critical single cell analytical technique in biomedical science, the technology of flow cytometry associated cell sorting is equally important. Physical separation of cells analyzed by flow cytometry was recognized as an important goal even in the field’s beginning, and many of the earliest cytometers were also cell sorters. Isolation of cells based on flow cytometric analysis has formed the foundation of immune cell differentiation and development and continues to grow importance as techniques for genomic and proteomic analysis expand. This brief review will describe both the historical development and current state of cell sorting. The multiple mechanisms for cell sorters will be covered, and critical aspects of cell sorting will be discussed. Newer technologies for cell sorting including microfluidic technologies will also be considered.

## Introduction

Cell sorting (sometimes referred to as fluorescence-activated cell sorting or FACS^1^) is an important extension of flow cytometry (1). Non-sorting flow cytometers analyze cells but then discard them. Cell sorters use one of several mechanisms to physically separate cells into subpopulations based on the results of cytometric analysis (2). While other techniques such as magnetic bead separation can somewhat purify cells based on phenotype, cell sorters can rapidly separate any population that can be defined by flow cytometry. This permits cell separation based on highly complex phenotypic data. Cell sorters can achieve high levels of cell purity and can precisely count the number of cells sorted. They can also precisely transfer the sorted cells into tubes or plates. Single cell sorting for RNASeq and other genomic methods are now routinely employed for precise assessment of immune cell phenotype and function.

The original development of cell sorters occurred at essential the same time as the development of the flow cytometer. While early immunologists were interested in cellular phenotype the number of cell markers and fluorescent means to label them was very limited. Investigators therefore considered physical separation of cells based on light scatter and the small number of molecular markers available at the time to be just as critical it is today. One of the first practical flow cytometers was developed by Mac Fulwyler at Los Alamos National Laboratory and was based on a direct impedance flow cell rather than stream-in-air or cuvette systems prevalent today (3). This analyzer was a cell separation device as well as an analyzer since its goal was to separate differential cell populations for subsequent microscopic analysis. One of the first cell sorters based on laser-induced light scatter and fluorescence measurement was the Becton Dickinson FACS 1 (One) co-developed with Len and Leonora Herzenberg at Stanford University (4). Similar systems were developed by the Coulter Corporation, including the EPICS sorters (5). These systems used piezo-based oscillation to break a cell stream into droplets. This was similar to the mechanism for generating droplets in inkjet printing, and in fact was developed with the assistance of the same individuals working in printer technology (6). A mechanism for charging the stream prior to drop breakoff and electrostatic plates to divert droplets into collection tubes allowed rapid cell sorting that corresponded precisely to prior flow cytometric analysis. While higher pressure fluidics, faster electronics and digital data acquisition have improved these systems, most modern cell sorters rely on the same electrostatic technology that drove the original sorters. The earliest systems were used primarily to separate cell populations for subsequent cell culture; these experiments were crucial in defining the ontogeny of hematopoesis and immune cell development (7, 8). Early systems struggled to produce sufficient number of cells for early genomic and proteomic techniques such as Southern, Northern and Western blotting. While sorters have increased in collection rate, modern genomic and proteomic techniques now require far fewer cells, increasing the utility of even slower cell sorting systems. Cell sorters are now used for a variety of purposes, including isolating cell populations for subsequent genomic and proteomic analysis (9). The ability of modern genomic and proteomic techniques to use very small numbers of cells has tremendously enhanced the utility of cell sorting in biomedical and biological research.

### Electrostatic cell sorters

**Figure 1:**
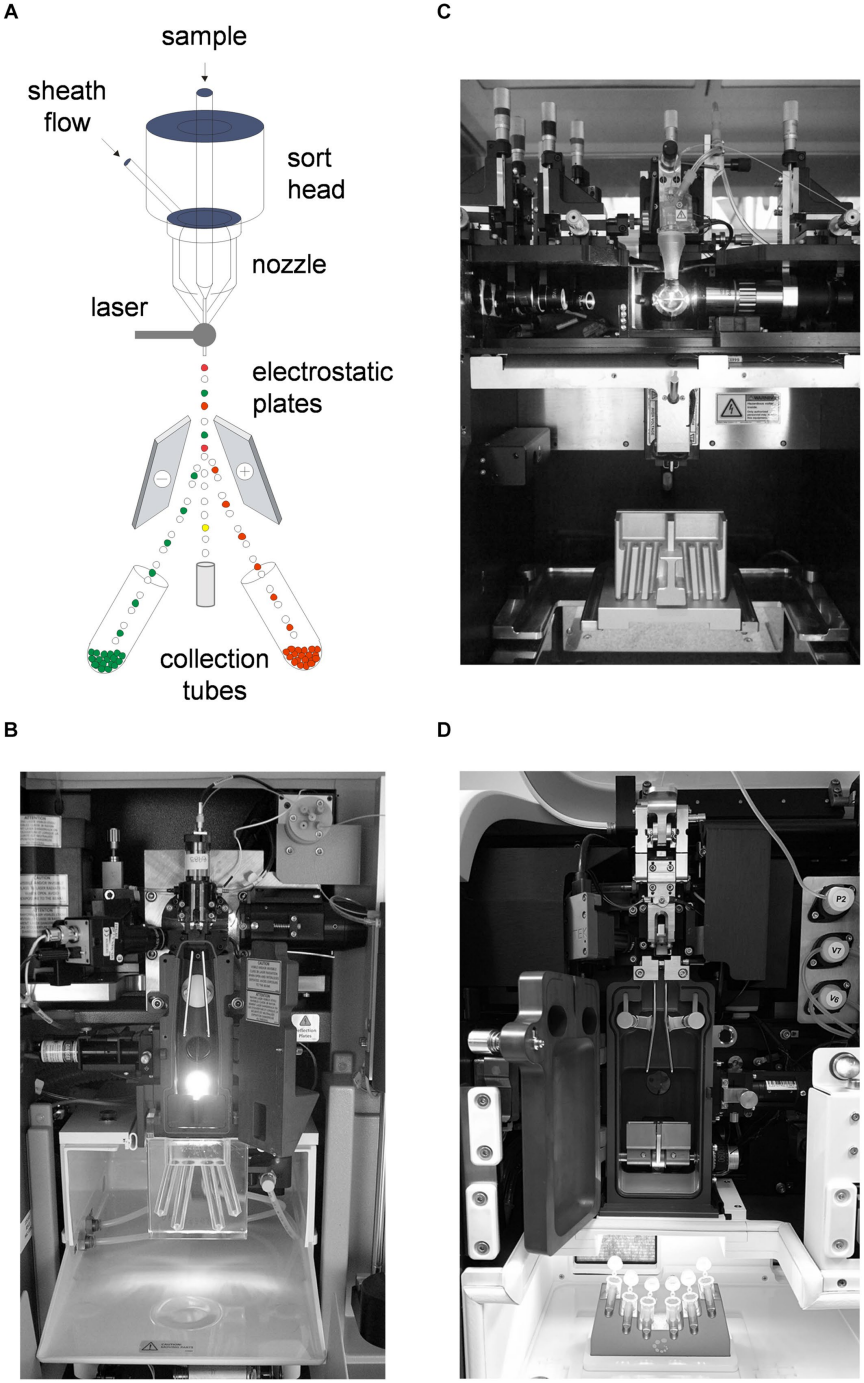
Electrostatic cell sorter systems. **(A)** Schematic of a jet-in-air cell sorter showing nozzle, laser intercept, stream and droplet formation, electrostatic deflection plates and collection tubes. **(B)** Interior of an Influx cell sorter, typical of a jet-in-air electrostatic system (BD Biosciences). **(C)** Interior of a FACSAria IIu cell sorter, typical of a hybrid cuvette electrostatic cell sorter (BD Biosciences). **(D)** Interior of an Aurora CS electrostatic cell sorter, typical of a spectral hybrid cuvette electrostatic system (Cytek Biosciences). Nozzles, electrostatic deflection plates and collection tubes are visible on all systems.

Most cell sorters still rely on piezo-driven droplet generation and electrostatic separation (5–7). Figure 1A shows a schematic of a jet-in-air electrostatic sorter. Cells are initially interrogated by one or more lasers just below a nozzle, where the hydrodynamically focused cells are ejected. The stream is simultaneously oscillated at a high frequency (in the tens of kilohertz range) to break it into droplets. The time required for a cell to pass from the laser intercept point to the last connected cell droplet is calculated, and the stream is then given a positive or negative charge based on the cell phenotype that the investigator wishes to sort. The drops then break off from the stream and are diverted into collection tubes using high-voltage electrostatic plates.

The interiors of several sorting systems are shown in Figure 1. All are based on the technology shown in Figure 1A, differing only in the presentation of cells to the instrument signal collection optics. Figure 1B shows a *jet-in-air system*, where the cells are ejected from a nozzle into the open air for laser interrogation (Influx cell sorter, BD Biosciences). The electrostatic plates and collection tubes are visible. Figure 1C shows a *hybrid cuvette system*, where the cells are first analyzed in a quartz cuvette similar to a non-sorting analyzer (FACSAria II cell sorter, BD Biosciences). The cells are then ejected into the open air for droplet formation. Cuvette systems have better fluorescence sensitivity than jet-in-air systems since the resulting fluorescent signals are more efficiently transmitted to the instrument optics and are now more common. Figure 1D shows another *hybrid cuvette system* on a spectral cell sorter (Aurora CS spectral sorter, Cytek Biosciences), which collects complete cell spectra rather than individual fluorescence bandwidths on conventional instruments. Spectral cytometry is rapidly gaining favor due to its ability to collect larger simultaneous numbers of fluorescent probe signals for high-precision cell analysis (10, 11). Older traditional systems and newer spectral systems nevertheless use the same cell sorting technology. Electrostatic systems can collect multiple cell populations simultaneously through differential stream charging, allowing collection of four or six populations simultaneously.

**Figure 2:**
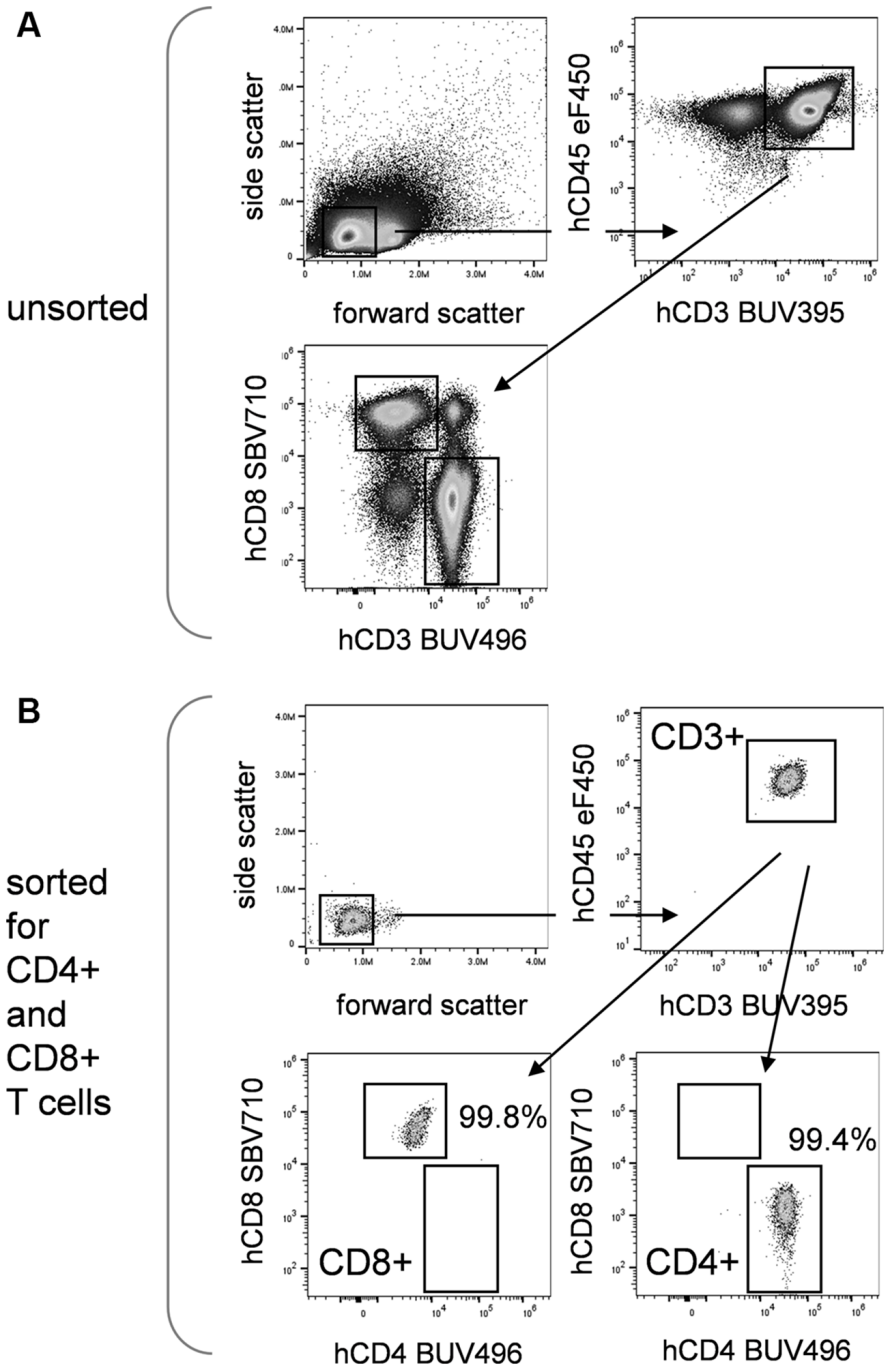
Cell sorting. Flow cytometric analysis and sorting of human peripheral blood cells (PBMCs, Veri-Cells, Biolegend) for leukocyte and T cell markers. **(A)** Analysis of unseparated PBMCs for forward and side scatter, CD3 versus CD45 expression gated on scatter, and CD4 and CD8 expression gated on CD45 + CD3+ cells. Sorting was then carried out for small cells based on forward versus side scatter, CD3 and CD45 positive expression, and CD4 or CD8 positive expression using the indicated gates. **(B)** Analysis of sorted PBMCs using the indicated gates. Cells have been purified for small cells expressing CD3 and CD45, and either CD8 or CD4. Purity for sorted CD8 and CD4 populations exceeded 99%.

Modern electrostatic cell sorters can sort nearly 30,000 total events per second with purities exceeding 95%, although these values are very dependent on cell type, initial prevalence of the cell type to be sorted, and condition of the sample. An example is shown in Figure 2, where human T cells have been sorted to high purity from peripheral blood mononuclear cells based on multiple phenotyping markers. Figure 2A shows the unsorted cells labeled for several leukocyte and T cell markers. Figure 2B shows reanalysis of sorted CD4+ and CD8+ T cells, which were separated using all the cell labels. Very rare cells such as stem cells can also be sorted in this way, although purities and cell yields may be less than for more common populations. Cells can be collected into tubes, microtiter plates, PCR tubes or genomic analysis cartridges, allowing them to be used for subsequent outgrowth experiments or genomic and proteomic analysis. Collection of small numbers of cells including single cells for genomic analysis is now routine, although the Poisson statistics of cell distribution in a stream does not give this method outstanding precision. Microfluidic-based sorting systems give better precision for this application, although at slower speeds (12, 13).

### Critical factors

There are many critical factors that must be taken into account when using cell sorting for cell separation. The nozzle or cuvette diameter, sheath and sample pressure, frequency and period of the stream drop generation and the distance/time for the cell to move from the laser intercept to the point of drop generation are all conditions that can be modified and must be taken into account when setting up a sort (1). Large cells, rare cells and cells prone to adherence all present special challenges to collection purity and efficiency. The high pressures used in cell sorting can also damage fragile cells. Cell sorters can be operated to emphasize cell purity, but this can sacrifice yield; high yield sorting will also sacrifice cell purity (2). Rare stem cells, for example, may be collectable only with sorting that maximizes yield, meaning the resulting collection will be less pure (14). Larger cells will require a larger nozzle (and hence a larger drop size) and lower sheath and sample pressures to allow separation without disruption of the sort streams. Cell sorting is often combined with prior enrichment techniques such as magnetic separation to enhance purity and yield and reduce the overall time of the sort. Cells collected for subsequent culture require both a sterilized instrument and post-sort precautions such as culture in antibiotics to prevent contamination. Electrostatic sorting operates to a large degree in the open air, requiring containment systems for samples that may pose a biohazard (15).

### Microfluidic based cell sorters

**Figure 3:**
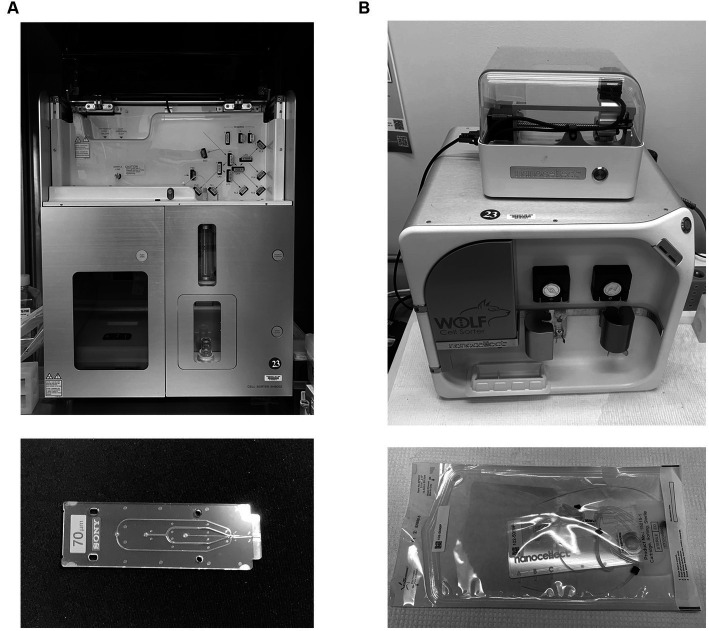
Microfluidic-based cell sorting systems. **(A)** A Sony SH800 cell sorter system (Sony Biotechnology), which employs a microfluidic chip for cell analysis and stream formation, but still relies on stream charging and electrostatic plate separation. **(B)** A Wolf cell sorter (Nanocellect Biomedical), which both analyzes and sorts using a microfluidic chip. Chips are shown below each instrument.

Dramatic improvements in fabrication of microfluidic devices have also led to the development of microfluidic-based flow cytometers and cell sorters (12, 13, 16). These systems use a chip with microfluidic channels and mechanical gates or air pressure to divert a cell from a central stream, again following upstream analysis. Microfluidic-based systems have several advantages over electrostatic systems. Electrostatic systems employ high air pressure to generate sheath-contained sample streams; this pressure can damage fragile cells. Microfluidic based systems operate at lower pressure and sort more gently (17). Cell positioning is also more exact in microfluidic systems, allowing more precise collection of single cells. Microfluidic systems are however much slower than electrostatic systems, and at this writing do not yet employ the large number of lasers and detectors found on traditional sorters (16–18). They also collect fewer populations, often only one. Figure 3A shows a Sony SH800 cell sorter (Sony Biotechnology); while a hybrid cuvette system, it uses a plastic microfluidic chip for both stream formation and cell interrogation, different from the ceramic nozzles or quartz cuvettes typically employed in electrostatic systems. Figure 3B shows a Wolf cell sorter (Nanocellect Biomedical) which uses a disposable microfluidic chip for both analysis and separation, making it a true microfluidic-based sorter. The Tyto (Miltenyi Biotec) employs a high-speed mechanical gating system to divert a single population into a collection reservoir via a disposable microfluidic chamber system. The ability to use disposable sorting chambers has led to high-throughput systems that are proposed for sorting cells for therapeutic purposes. Microfluidic-based cell sorter from On-Chip Biotechnologies (PHC Corporation) have been used to sort larger biological objects using a disposable fluidic chip system. The Cytonome systems use multiple parallel microfluidic systems to sort large numbers of cells using single-use cartridge. While cell sorting has until recently been used largely for research applications, recent efforts to purify cells for *in vivo* clinical applications (such as stem cell separation bone marrow transplant, CAR-T and other cell-based therapies) are focusing on microfluidic based systems for their ability to use disposable closed cartridge-based systems (19). However, cell sorting for subsequent clinical analysis of mutations is now much more common and takes advantage of the ability to sort small cell numbers and individual cells.

### Other technologies

Piezo-driven collection needles that can insert into a cell stream without droplet generation (not to be confused with piezo droplet formation in electrostatic sorters) have appeared in a few commercial cell sorters, including the discontinued BD Biosciences FACSort and FACSCalibur analyzers, and the Partec/Sysmex systems. The ability for these systems to be fully enclosed (in contrast to electrostatic sorters, which operate in the open air and need aerosol precautions) has made these systems attractive for analysis in biohazard environments. However, these systems sort at very low sort rates, and have been largely supplanted by microfluidic-based systems. The use of acoustic fields to generate single cell streams has been employed as an alternative to hydrodynamic focusing by some cytometers; objects of different sizes can be acoustically focused, potentially allowing downstream capture (18, 20). While a sorter using this technology has yet to be built, analyzers including the Thermo Fisher Life Scientific Attune NxT and the BennuBio Velocyt employ acoustic fields for cell stream focusing. While cell sorting technology is now relatively mature, a limit has been reached on the maximum throughput for traditional methods. New technologies for cell sorting that may be able to overcome these limitations are therefore of tremendous interest.

## Author contributions

WT: Conceptualization, Writing – original draft.

## Footnotes

^1^Fluorescence activated cell sorting or FACS is a registered trademark of Becton Dickinson Immunocytometry Systems (BD Biosciences).
